# The Pharmacists in Primary Care Network Program: Practice innovation in British Columbia

**DOI:** 10.1177/17151635251353242

**Published:** 2025-07-23

**Authors:** Peter J. Zed, Peter S. Loewen, Anita I. Kapanen, Arwa Nemir, Anupama Salil

**Affiliations:** Faculty of Pharmaceutical Sciences, University of British Columbia, Vancouver, BC; Department of Emergency Medicine, Faculty of Medicine, University of British Columbia, Vancouver, BC; Faculty of Pharmaceutical Sciences, University of British Columbia, Vancouver, BC; Faculty of Pharmaceutical Sciences, University of British Columbia, Vancouver, BC; Faculty of Pharmaceutical Sciences, University of British Columbia, Vancouver, BC; Faculty of Pharmaceutical Sciences, University of British Columbia, Vancouver, BC

## Introduction

The pharmacist profession is ever-evolving to align pharmacist roles and scope of practice with the needs of patients and society. Pharmacists have the training and skills to meet these needs through the provision of comprehensive medication management (CMM),^1^ which includes patient assessment and identification, prioritization of drug therapy problems (DTPs), collaborative care plans, prescribing, and follow-up to optimize health outcomes for patients.

The benefits to patients who receive care in a variety of settings from a team of health care providers that includes a pharmacist are well-established.^2,3^ Such integration is prevalent in hospital settings but is more nascent in primary care in Canada than in other countries.^4,5^ Successful integration of pharmacists into primary health care models has been well-established in England, Ireland, and Australia.^6-8^ In Canada, pharmacist integration into primary care teams has been supported by pharmacists and family physicians.^9,10^ The first integration of pharmacists into family health teams in primary care occurred in Ontario, where it continues to be the most established.^11,12^ This was followed by similar programs in Alberta and Quebec.^13,14^ Integration of pharmacists into team-based primary care in other provinces has evolved with varying pace, approach, and models.^5^

In June of 2018, the British Columbia (BC) government announced new funding for a program to integrate pharmacists into team-based primary care practices.^15,16^ This was a component of a large-scale effort by the BC Ministry of Health (the Ministry) to enhance primary care delivery across the province. The goal was to establish primary care networks (PCNs) to deliver interprofessional team-based care.^17^ In preparation for this system change, a white paper was published to support the broad system changes that would affect all pharmacists and to support the optimization of pharmacist-delivered care throughout the BC health care system.^5^

## The BC Pharmacists in PCN Program

In BC, the Pharmacists in PCN Program (the program) began in 2020 and integrated primary care clinical pharmacists (PCCPs) as members of interprofessional teams (IPTs), which consisted, depending on the PCN, of family physicians, nurse practitioners, pharmacists, social workers, physiotherapists, occupational therapists, dietitians, and counsellors. Pharmacists were expected to provide CMM and offer their expertise in drug therapy decision-making for complex patients. The goals of pharmacist participation in the PCNs were to reduce DTPs; reduce unnecessary drug use and negative drug therapy consequences; increase patient, family, caregiver, physician, pharmacist, and health care team satisfaction; and increase information sharing and collaboration between pharmacists and other members of the patient care team within the PCN community, including community pharmacists.

### Governance

The program was conceived and led by the University of British Columbia (UBC) Faculty of Pharmaceutical Sciences (the faculty) as a major initiative within its Practice Innovation portfolio. UBC led the program’s implementation and oversaw its ongoing operations provincially. UBC program team members were responsible for orientating and training all PCCPs, supporting PCCP work activities, and providing quality assurance. UBC was also responsible for developing and executing an evaluation of the program conducted by an evaluation team with members independent of the program implementation team.

UBC collaborated with many stakeholders throughout implementation, primarily regional health authorities, PCNs, and the Pharmaceutical, Laboratory, and Blood Services and Primary Care divisions of the Ministry. The Ministry provided funding for program implementation and evaluation. Health authorities were the employers of PCCPs and were responsible for recruitment and maintenance of the employment relations with PCCPs. PCNs provided local context and were responsible for supporting and enabling PCCPs to integrate into existing structures, processes, and care teams within their respective PCN communities.

**Figure 1:**
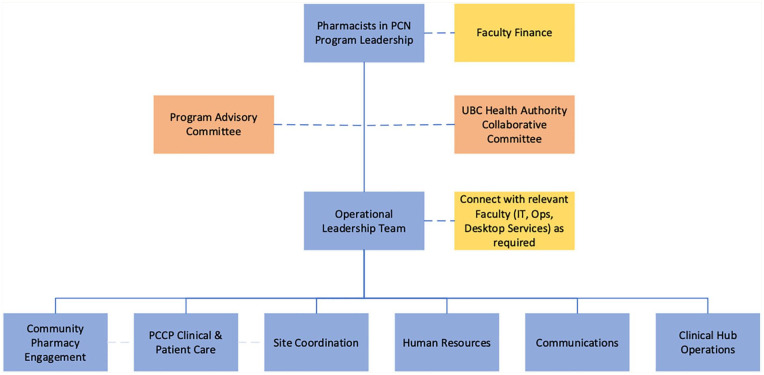
Pharmacists in PCN Program governance IT, information technology; Ops, operations; PCCP, primary care clinical pharmacist; PCN, primary care network; UBC, University of British Columbia.

The program was supported by 2 committees (Figure 1). A Program Advisory Committee was established to facilitate information sharing and collaboration between UBC, the Ministry, PCNs, and regional health authorities to inform how PCCPs could be best integrated and expanded in the health care system over the long-term. The UBC Health Authority Collaborative Committee was established to support the hiring and onboarding of PCCPs and to enable reasonably consistent processes for administering the PCCP employment relationship and performance standards across the health authorities.

### Program team and resources

**Figure 2:**
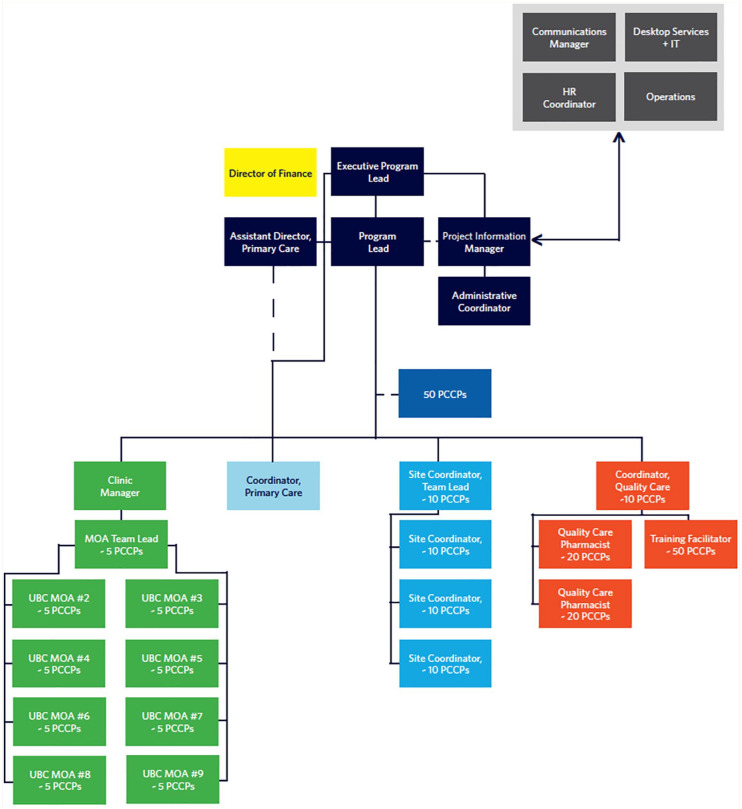
Pharmacists in PCN Program organizational structure HR, human resources; IT, information technology; MOA, medical office assistant; PCCP, primary care clinical pharmacist; PCN, primary care network; UBC, University of British Columbia.

The UBC program team consisted of an executive lead, program lead, program and information manager, primary care coordinator, and leads for quality care, site coordination, and medical office assistants (MOAs) (Figure 2). The quality care team consisted of a coordinator, 2 pharmacists, and a training facilitator who supported PCCP training and onboarding, learning plans, quality assurance, clinical resources, and development of a PCCP Community of Practice. The site coordination team consisted of the lead and 3 coordinators (~1 per 10 PCCPs) responsible for local onboarding, workflows, relationship building between the program and PCNs, and issues management. Nine MOAs (~1 per 5 PCCPs) supported PCCPs with referral processing, scheduling appointments, information sharing, and documentation in the program electronic medical record (EMR) and PharmaNet.^18^ The primary care coordinator was responsible for leading community pharmacy engagement activities to support the awareness and opportunities for PCCPs to collaborate on the shared care of mutual patients.

The faculty supported human resources, finances, communications, and information technology. A Community Engagement Working Group supported and enabled the shared care of mutual patients between PCCPs and pharmacists practicing in different health care settings and promoted engagement with other groups, including other health care providers and community groups. The program used a dedicated, centralized EMR (an open-source, modified version of OSCAR, originally developed by McMaster University), in which documentation for all patient appointments was maintained. The program team and PCCPs were enabled with all equipment, supplies, and technology support required for their practice.

The program also developed the Pharmacists Aligned in Shared Care Teams (PACT)^19,20^ education program, which was offered to all BC pharmacists to support the evolving practice changes in the province and to promote intraprofessional collaboration between PCCPs and other pharmacists more effectively.

### Program structure and approach

After the Ministry determined PCN readiness for team-based care, each PCN was allocated resources and approval to hire 1 PCCP. Despite the preference for a consistent team-based care model, unique features of each PCN meant that the program team approached integration with flexibility to optimize the PCCP role in the local context and efficiently integrate the PCCP into the PCN team and local community. Factors considered for each PCN included the preferred model of care (i.e., co-location, hub and spoke, or blended), service delivery mode (i.e., in-person, telephone, or video), referral and scheduling processes, care mapping, case conferences, and sharing documentation (e.g., PCN EMR, eFax, Clinical Data eXchange [CDX]^21^). The program was designed on a model of full co-location of the PCCPs physically practicing within PCN clinics alongside other members of the IPT; however, most PCNs used a hub and spoke model, in which the PCCPs and other members of the IPT worked in a decentralized location, or a blended model in which PCCPs divided their time between working in co-location and within hubs.

Most patients were seen by a PCCP following a referral from a family physician or nurse practitioner. UBC MOAs scheduled the appointments, which were 60 minutes for initial appointments and up to 30 minutes for follow-up appointments. Appointments took place in-person or using telehealth technology by telephone or via Zoom. The first period of program implementation occurred during the COVID-19 pandemic, so types of appointments were dictated by provincial guidelines on how patients were permitted to access their primary health care team. Overall, PCCPs were expected to spend 80% of their time in direct patient care activities (appointments, case conferences, rounds, discussions with care team, and documentation) and 20% of their time on other activities (non-patient-specific meetings, outreach, education delivery, information sharing, and professional development).

The program was funded to integrate up to 50 PCCPs over the 3-year implementation period: 20 PCCPs in year 1 and 30 more in years 2 and 3.

### Program evaluation

Program evaluation occurred in parallel with program implementation. The evaluation used a logic model, evaluation framework, and a series of evaluation questions to understand how the program affected user experiences (i.e., patients, pharmacists, health authority representatives, PCN administrators, PCN IPT members, and community pharmacists) and quality of care when receiving care from a PCCP.

Short- to intermediate-term implementation outcomes explored 5 domains: (1) program infrastructure; (2) site coordination and PCCP integration; (3) service demand and integration; (4) quality assurance; and (5) community/hospital pharmacy engagement. The 3-year outcomes associated with quality of care were evaluated in 4 domains: (1) patient outcomes; (2) patient experience; (3) interprofessional collaboration; and (4) intraprofessional collaboration.

Herein we provide a brief description of the program implementation evaluation. More detailed perspectives and experiences of implementation, as well as quality of care outcomes, will be published separately.

## Implementation outcomes

The program was implemented over the 3-year period between October 1, 2020 and September 30, 2023. Fifty-nine PCCPs provided care in 47 PCNs over the implementation period. The median (range) duration that a PCN had a PCCP integrated was 82 weeks (9–134). The models of care among the 47 PCNs at the end of the implementation period were blended (37; 78.7%), hub and spoke (7; 14.9%), and co-location (3; 6.4%). Among PCCPs in a blended model, only 20% of their patient care time was spent in co-location within PCN clinics, whereas the majority of their clinical care time was provided while in the hub and spoke model.

Hiring PCCPs was challenging and included several steps such as time to receive Ministry approval to proceed with hiring a PCCP, approval to job posting, job posting to hire, and hire to starting the position. Overall, the median (range) time from Ministry approval to starting the position was 248 days (90–801). Once started, PCCPs underwent orientation and onboarding, which delayed seeing their first patient by a median of 29 days (5–74).

PCCPs conducted 24,098 patient appointments with 7456 unique patients. Patients had a mean (SD) age of 67.5 years (16.7), 59% were female, 79.0% were receiving ≥5 medications, and 54.0% were receiving >10 medications. Patient appointments by health authority were Island Health (36.7%), Interior Health (21.2%), Fraser Health (18.8%), Vancouver Coastal Health (16.4%), and Northern Health (6.8%). Patient appointments slowly increased throughout the 3-year implementation attributable to both increasing number of PCCPs hired as well as the experience and effective integration of the PCCP into IPTs over time.

PCCPs identified 30,085 DTPs that required intervention, monitoring, or education. Classification of DTPs included need to add drug (23.8%), unnecessary drug (17.6%), adverse drug reaction (17.6%), non-adherence (11.7%), drug dose too low (10.7%), drug change required (9.5%), and drug dose too high (9.1%).

## Conclusion

The 3-year implementation of the Pharmacists in PCN Program was supported by the combination of effective centralized administration by UBC and collaboration between UBC, health authorities, and PCNs. The program successfully integrated the first cohort of PCCPs in team-based care and established a foundation for longer term growth and sustainability. Following the successful 3-year implementation period led by UBC, the program operations were transitioned to the Provincial Health Services Authority (PHSA) in October 2023, which continues to provide centralized provincial support in collaboration with the regional health authorities, PCNs, and other community partners and stakeholders.

Experiences gained from program evaluation will be further described in a series of publications, which will share the experiences of program implementation and pharmacist integration into team-based primary care in BC. In addition, user experiences from patients, pharmacists, PCN administrators, and PCN IPT members will be described. We will also report on quality of care outcomes for key performance indicators established for pharmacy practice and primary care pharmacotherapy. ■
